# Editorial: Omics of Human Aging and Longevity in the Post Genome Era: From Single Biomarkers to Systems Biology Approaches

**DOI:** 10.3389/fgene.2022.913531

**Published:** 2022-05-02

**Authors:** Serena Dato, Ignazio S. Piras

**Affiliations:** ^1^ Department of Biology, Ecology and Earth Sciences, University of Calabria, Rende, Italy; ^2^ Neurogenomics Division, The Translational Genomics Research Institute, Phoenix, AZ, United States

**Keywords:** genetics, aging, omics, dataset, database, data integration

Aging is a complex and heterogeneous phenotype influenced by a combination of genetic and environmental factors, which modulate the individual chance of living longer. Decades of research demonstrated that the genetic determination in the way we age is due to a large number of genetic variants with small effects. These variants are localized in key interrelated pathways with few crucial hubs interconnecting different biological routes and modulated by the environment. The discovery of the large portion of phenotypic variance in the quality of aging is complicated by the biological complexity of the underlying molecular mechanisms; it is now clear that, after decades of reductionist studies, a full understanding of aging determinants requires a holistic approach, moving from the single variant association study to the fine analysis of the integrated networks implied in age-related diseases (ARDs). The availability of “omics data” has increased in the last years due to high-throughput technologies, and it promises to revolutionize the identification of biomarkers through an understanding of aging as a whole system and the development of strategies to improve health at old age.

To help the reader take stock of the situation and point to future perspectives of the field, in this Special Issue we collected a total of 6 articles, including three reviews and three original articles, covering different determinants of the aging process from genomics to environmental factors. Among the topics discussed, particular attention has been given to the use of public datasets. In fact, despite a large number of genomics datasets currently available, as a result of the development of high-throughput technologies, the integration and interpretation of multi-omics results are still challenging. Additionally, in spite of the large number of longitudinal studies carried out in the field, which potentially represent a powerful tool to investigate the occurrence of aging changes, data sharing among groups is still limited. In their review, Dato et al. investigated the multi-omics bioinformatics approaches applied to the aging process. Among the most powerful and innovative methods of multi-omics integration, they underlined the use of the tensor decomposition, applicable to uncover hidden relationships and also for results visualization. Other innovative methods are machine learning and deep neural networks. Additionally, the authors provide a list of useful resources including datasets related to aging, such as AgeFactDB, a repository that collects and integrate age phenotype data, and NeuroMuscleDB, a database of genes associated with muscle development, neuromuscular diseases, aging, and neurodegeneration. Finally, they propose a few recommendations, such as prioritizing diversity (e.g., including multiple types of data and from different populations), and investing in data sharing. Focusing on the subject of public data, two articles published on this topic were conducted using exclusively genomic datasets obtained from public repositories. Napolioni et al., leveraging data from large Genome-Wide Association Studies (GWAS) across several ethnic groups, demonstrated the association of recent consanguinity and autozygosity on the risk of late-onset Alzheimer’s Disease (LOAD), independently of educational attainment and *APOE* genotypes. Additionally, they were able to detect a rare recessive variant in a cohort of consanguineous LOAD patients and controls, located in the *RPH3AL* gene. A possible mechanism proposed, according to previous studies, is related to the causal role of blood pressure and cholesterol level on LOAD phenome. Podder et al. used existing data from different species, with the goal to discover new genes associated with aging, generating an interactive data portal to browse the results. Their investigation confirmed the role of pathways such as FoxO signaling, mTOR signaling, and autophagy in aging determination, shared among humans, mice, fruit flies, and worms. Interestingly, the target proteins of the FDA-approved drug rapamycin (inhibitor of mTOR) were conserved across all four species. This study confirms the importance of comparative genomics to unveil the mechanism underlying the aging process, as discussed by Treaster et al. in their review. They summarized the studies investigating the genetic background at the base of age-associated diseases and longevity across species, underlying as the use of comparative genomics, because of shared associated pathways across species, like those cited above, as well as the massive number of genomic datasets now available for a large number of organisms, can help to prioritize genes and markers to investigate novel genetic components of longevity in humans too. In particular, innovative methodologies of comparative genomics showed gene sets under constrained sequence evolution, which likely can represent important determinants of aging. Among the research articles, Mohammadnejad et al. applied an original regulatory network analysis on a large cohort of monozygotic twins, with the goal to evaluate the gene expression signatures associated with cognitive function. They identified five novel genes associated with cognition, like *APOBEC3G*, *H6PD*, *SLC45A1*, *GRIN3B*, and *PDE4D*, as well as dysregulation in ribosome function and focal adhesion as key pathways in neurodegeneration. Through transcription factor (TF) analysis they identified several regulons associated with cognitive function, some activated and others repressed. Furthermore, they investigated the significance of previously reported cognitive function-related TFs through a gene regulatory network analysis, demonstrating that their approach was able to detect important and meaningful differentially expressed genes and biological pathways implicated in cognitive function. Finally, the review of Misra focused on the chemical exposome, defined as the total of molecules to which the human body is exposed, affecting the internal homeostasis and perturbating the biological pathways. Examples of chemical exposomes are diet, drug use, smoking, and alcohol, but also nitrosamines, pesticides, and heavy metals. Over the past decade, the development of methods for cheminformatics allowed a deeper exploration of the chemical exposome from data collected using both animal models and human cohorts. The chemical exposome affects the aging process, specifically accelerating skin aging, shortening telomere length, and decreasing cognitive status, although not all the chemical exposomes harm the aging process, like selenium and crocin, demonstrated to have a beneficial effect. The paper discusses the open questions and the many challenges remaining to be addressed, such as the amount of contribution from the genome, how the human aging exposome can help to understand human health (e.g., metabolic syndromes, cancer, and neurodegenerative diseases), the existence of sex differences, the combination and effect of single chemicals on the aging process.

In conclusion, this Research Topic covered different aspects of the aging process, highlighting the importance of integrating the large amount of new data coming from innovative and original study designs in the Post Genome Era. If properly treated, the advancement of bioinformatics methodologies and the large availability of public data at the multi-omics level will make really possible the discovery of new molecular pathways associated with the aging process and potential molecular targets for aging-related diseases, representing a unique opportunity offered to biogerontologists today.

